# Functional Analysis of Neuronal MicroRNAs in *Caenorhabditis elegans* Dauer Formation by Combinational Genetics and Neuronal miRISC Immunoprecipitation

**DOI:** 10.1371/journal.pgen.1003592

**Published:** 2013-06-20

**Authors:** Minh T. Than, Brian A. Kudlow, Min Han

**Affiliations:** Howard Hughes Medical Institute and Department of Molecular, Cellular, and Developmental Biology of University of Colorado, Boulder, Colorado, United States of America; Massachusetts General Hospital and Harvard Medical School, United States of America

## Abstract

Identifying the physiological functions of microRNAs (miRNAs) is often challenging because miRNAs commonly impact gene expression under specific physiological conditions through complex miRNA::mRNA interaction networks and in coordination with other means of gene regulation, such as transcriptional regulation and protein degradation. Such complexity creates difficulties in dissecting miRNA functions through traditional genetic methods using individual miRNA mutations. To investigate the physiological functions of miRNAs in neurons, we combined a genetic “enhancer” approach complemented by biochemical analysis of neuronal miRNA-induced silencing complexes (miRISCs) in *C. elegans*. Total miRNA function can be compromised by mutating one of the two GW182 proteins (AIN-1), an important component of miRISC. We found that combining an *ain-1* mutation with a mutation in *unc-3*, a neuronal transcription factor, resulted in an inappropriate entrance into the stress-induced, alternative larval stage known as dauer, indicating a role of miRNAs in preventing aberrant dauer formation. Analysis of this genetic interaction suggests that neuronal miRNAs perform such a role partly by regulating endogenous cyclic guanosine monophosphate (cGMP) signaling, potentially influencing two other dauer-regulating pathways. Through tissue-specific immunoprecipitations of miRISC, we identified miRNAs and their likely target mRNAs within neuronal tissue. We verified the biological relevance of several of these miRNAs and found that many miRNAs likely regulate dauer formation through multiple dauer-related targets. Further analysis of target mRNAs suggests potential miRNA involvement in various neuronal processes, but the importance of these miRNA::mRNA interactions remains unclear. Finally, we found that neuronal genes may be more highly regulated by miRNAs than intestinal genes. Overall, our study identifies miRNAs and their targets, and a physiological function of these miRNAs in neurons. It also suggests that compromising other aspects of gene expression, along with miRISC, can be an effective approach to reveal miRNA functions in specific tissues under specific physiological conditions.

## Author Summary

MicroRNAs (miRNAs) are important in the regulation of gene expression and are present in many organisms. To identify specific biological processes that are regulated by miRNAs, we disturbed total miRNA function under a certain genetic background and searched for defects. Interestingly, we found a prominent developmental defect that was dependent on a mutation in another gene involved in regulating transcription in neurons. Thus, by compromising two different aspects of gene regulation, we were able to identify a specific biological function of miRNAs. By investigating this defect, we determined that neuronal miRNAs likely function to help modulate cyclic guanosine monophosphate signaling. We then took a systematic approach and identified many miRNAs and genes that are likely to be regulated by neuronal miRNAs, and in doing so, we found genes involved in the initial defect. Additionally, we found many other genes, and show that genes expressed in neurons seem to be more regulated by miRNAs than genes in the intestine. Through our study, we identify a biological function of neuronal miRNAs and provide data that will help in identifying other important, novel, and exciting roles of this important class of small RNAs.

## Introduction

Classical genetics have uncovered important developmental functions of numerous microRNAs (miRNAs). However, these approaches have been limited in understanding other physiological functions of this extensive class of regulatory RNAs, partly because many miRNAs are dispensable under favorable, non-stressful conditions [1]. Particularly, the elimination of many miRNA families resulting in few apparent defects suggests that the nature of miRNA regulation is much more complex [2]. This complexity potentially arises from redundancy and coordination between multiple aspects of gene expression, ensuring robust modulation and control over developmental and non-developmental processes. Thus, compromising single miRNAs or even miRNA families may have limited effects on the entire regulatory network, and therefore would be a challenging approach to elucidating the biological functions of miRNAs.

We, and others, have found that compromising miRISC and total miRNA function helps to reveal processes sensitive to miRNA regulation [3], [4], [5]. Thus by compromising either Argonaute or GW182 function, miRNA-related processes can be identified [5]. To this end, compromising other regulatory mechanisms, in combination with miRISC, may further influence regulatory networks to reveal unknown physiological functions of miRNAs. In order to understand the physiological functions of miRNAs and their roles within complex regulatory networks, we investigated a genetic interaction between a neuronal transcription factor, UNC-3, and miRISC. UNC-3 is a conserved COE (Collier/Olf-1/Early B-cell Factor) transcription factor that is important in controlling motor neuron development as well as the identity of ASI chemosensory neurons that have been shown to play critical roles in dauer development [6], [7], [8]. Through investigating this complex genetic interaction, we found that miRNAs largely function in neurons to exert control over dauer development. The process of dauer development is highly regulated through multiple signaling pathways, and the robustness of these pathways is further reinforced through miRNA regulation to ensure ideal induction of dauer development.

Complementary to this genetic approach, we further identified miRNAs and miRNA-targets within neuronal tissue. In doing so, we found individual miRNAs and potential targets that modulate dauer formation. More broadly, our analysis suggests that neuronal miRNAs can modulate a wide range of neuronal processes and activities, including dauer formation.

## Results

### Neuronal miRNAs regulate dauer formation in coordination with UNC-3

**Figure 1:**
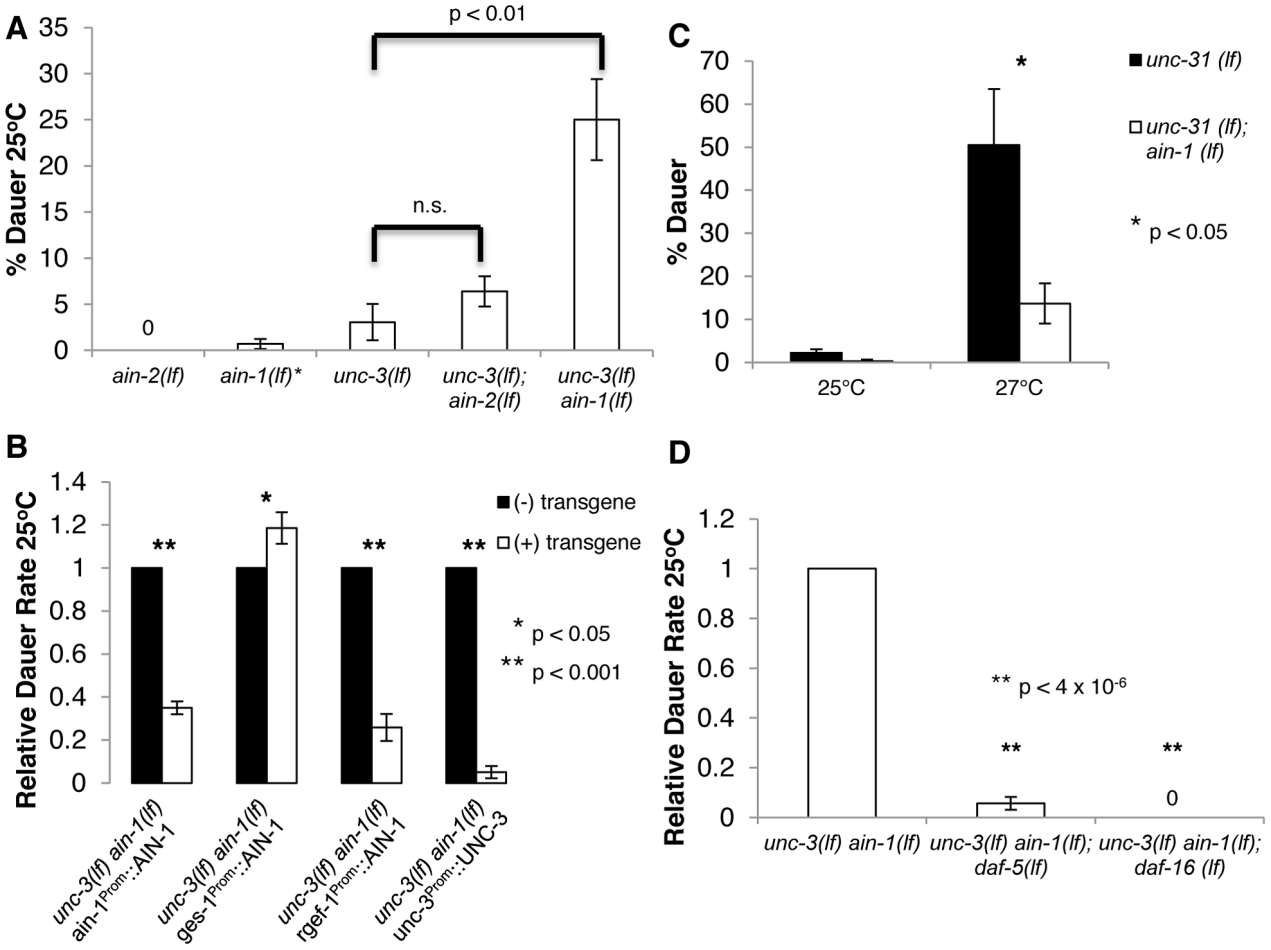
Neuronal miRNAs act in parallel to UNC-3 to repress undesired dauer formation by potentially affecting IIS and TGF-β signaling. **A–D.** **A.** Bar graph representing the percent of dauer progeny of indicated genotypes. * indicates that *ain-1(lf)* mutants were not tested in parallel to these specific experiments. Alleles used are *ain-1(ku322 lf), unc-3(e151 lf), and ain-2(tm2432 lf)* **B.** Relative dauer rate of *unc-3(e151 lf) ain-1(ku322 lf)* transgenic mutants carrying the designated extrachromosomal array. Relative dauer rate was determined by normalizing the percent of dauer progeny for transgenic and non-transgenic animals by the percent of dauer progeny of non-transgenic progeny on the same plate. This was done to account for plate-to-plate variation. The AIN-1 protein in each transgene was translationally fused to GFP. **C.** *ain-1(tm3681 lf)* mutation represses aberrant dauer formation of *unc-31(e928 lf)* at 27°C. **D.** *daf-5(e1386 lf)* and *daf-16(mu86 lf)* suppress aberrant dauer formation phenotype of *unc-3(e151 lf) ain-1(ku322 lf)*. The percent of dauer progeny is shown relative to the original *unc-3(lf) ain-1(lf)* mutant. Relative dauer rate was used to account for day-to-day variation. Error bars represent S.E.M. from at least three biological replicates. A student's t-test was used to determine statistical significance for all experiments.

UNC-3 has been indicated to affect dauer formation through multiple mechanisms. *unc-3(lf)* mutants show decreased expression of DAF-7/TGF-β, a major regulator of dauer formation [7]. At 15°C, neither *unc-3(lf)* nor *daf-7(e1372ts)* single mutants form a high percentage of constitutive dauers. However, at 15°C, *unc-3(lf); daf-7(e1372ts)* double mutants have a stronger constitutive dauer (Daf-c) phenotype than *unc-3(lf)* or *daf-7(e1372ts)* mutants. Thus, in addition to influencing TGF-β signaling, *unc-3(lf)* mutants also have TGF-β independent, dauer signaling outputs [9]. Despite influencing multiple aspects of dauer signaling, only a low percentage of *unc-3(lf)* animals form constitutive dauers at 25°C (Figure 1A).

AIN-1 and AIN-2 are two *Caenorhabditis elegans* (*C. elegans*) GW182 proteins that act semi-redundantly for miRNA-mediated gene silencing [10], [11]. We made an *unc-3(e151 lf) ain-1(ku322 lf)* double mutant and observed that double mutant animals formed more dauers than the *unc-3(lf)* mutant at 25°C, while *ain-1(lf)* alone does not display obvious defects in dauer formation (Figure 1A). The rate of dauer formation of *unc-3(lf) ain-1(lf)* mutants can be significantly reduced when a functional AIN-1::GFP or UNC-3 is expressed through an extra-chromosomal array (Figure 1B). These results suggest that both *unc-3(lf)* and *ain-1(lf)*, not a linked mutation, are responsible for the partial dauer-constitutive (Daf-c) phenotype of *unc-3(lf) ain-1(lf)* animals. This synergistic Daf-c phenotype suggests that miRNAs play an important role in modulating dauer responses and at least some of these functions are UNC-3 independent. Because *ain-1(ku322 lf); ain-2(tm1863 rf)* double mutants, which further compromise miRNA function, are not Daf-c, miRNAs appear to act in concert with multiple mechanisms to provide robustness to the regulatory system. However, we did not observe a strong increase in the rate of dauer formation in the *unc-3(e151); ain-2(tm2432 lf)* mutant, potentially suggesting that AIN-1 containing miRISC complexes play a more prominent role in modulating dauer formation (Figure 1A and Figure S1A).

To determine where miRNAs execute this function, we restricted the spatial expression of the AIN-1::GFP transgene in *unc-3(lf) ain-1(lf)* animals. Neuronal expression and intestinal expression were accomplished through *rgef-1* and *ges-1* promoters, as described previously [12], [13]. Expression of either neuronal AIN-1::GFP or AIN-2::GFP in the *unc-3(lf) ain-1(lf)* mutant was found to be sufficient to reduce the rate of abnormal dauer formation (Figure 1B and Figure S1B), indicating that neuronal miRNAs are largely responsible for the observed role of miRNAs in repressing dauer formation.

In contrast, we found that intestinally expressed AIN-1::GFP was not only insufficient to suppress the Daf-c defect, but also slightly increased the rate of dauer formation (Figure 1B). This intriguing result suggested that intestinal miRNAs may have a role in promoting dauer formation, which is contrary to the repressive role of neuronal miRNAs observed in *unc-3(lf) ain-1(lf)* animals. The potential role of promoting dauer formation is consistent with our previous finding that intestinal miRNAs repress activities of the insulin/IGF-1 signaling (IIS) pathway and the finding by others that miRNAs repress IIS signaling for longevity related functions [3], [14]. In order to confirm the potential role of intestinal miRNAs in promoting dauer formation, we tested the relationship of UNC-31 and AIN-1. UNC-31 is involved in insulin secretion and signaling. Interestingly, it was previously shown that *unc-31(lf); unc-3(lf)* mutants have a strong daf-c phenotype [9]. We found that *unc-31(e928 lf); ain-1(tm3681 lf)* double mutants formed dauers far less frequently than the *unc-31(e928 lf)* single mutant (Figure 1C), which is consistent with the idea that miRNAs could also play a role in promoting dauer formation and not simply repressing dauer formation, revealing a highly complex role of miRNAs in dauer-related signaling. To further test whether this dauer-promoting function was intestinal, we expressed an AIN-1::GFP fusion in the intestine of *unc-31(e928 lf); ain-1(tm3681 lf)* animals. We found that restoring AIN-1 in the intestine increased the rate of dauer formation, partially rescuing the *unc-31(e928 lf); ain-1(tm3681 lf)* phenotype (Figure S1C). There are likely other tissues involved as there was not complete rescue. Nonetheless, this result supports a role for intestinal miRNA function in promoting dauer formation. However, this role of intestinal miRNAs seems to be weak, as we see very slight increases (5–15%) of overall dauer formation in transgenic animals expressing AIN-1::GFP in the intestine.

Altogether, these results suggest that miRNAs within different tissues can exert opposing effects on biological processes. Furthermore, loss of neuronal AIN-1 appears to overcome the loss of intestinal AIN-1, resulting in an increased rate of dauer formation.

### The dauer constitutive phenotype of *unc-3(lf) ain-1(lf)* mutants is suppressed by constitutive DAF-7/TGF-β and IIS signaling

TGF-β and Insulin/IGF-1 signaling (IIS) are two well-known signaling cascades that regulate dauer formation [15]. Moreover, the release of insulin-like and TGF-β signaling ligands is predominantly neuronal and serves as a means to link environmental cues to development and metabolism. Our data suggest that neuronal miRNAs regulate dauer formation; because of the neural component of these pathways, we tested the dependence of these signaling cascades on our mutant phenotype.

To test the dependence on TGF-β signaling, we compared *unc-3(lf) ain-1(lf)* mutants to *unc-3(lf) ain-1(lf); daf-5 (e1386 lf)* triple mutants. DAF-5 is a member of the SNO/SKI superfamily and is antagonized by DAF-7/TGF-β signaling. While mutations in DAF-5 completely suppress the Daf-c phenotype of defective TGF-β signaling [16], loss of DAF-5 does not completely suppress the constitutive dauer formation of *daf-2*/IIS-R IIS mutants or *daf-11*/guanylate cyclase cGMP mutants [17], [18]. We found that a *daf-5(lf)* mutation effectively suppressed the abnormal dauer formation defect of *unc-3(lf) ain-1(lf)* mutants at 25°C (Figure 1D). This observation suggests that the manifestation of the *unc-3(lf) ain-1(lf)* mutant defect is dependent upon compromised TGF-β signaling for dauer formation.

Similarly to DAF-5 and TGF-β, DAF-16/FOXO is a negative factor downstream of IIS and *daf-16(lf)* can completely suppress Daf-c phenotypes of defects in IIS. However, *daf-16(lf)* is unable to completely suppress the Daf-c phenotype from TGF-β signaling mutants and *daf-11* mutants [17], [18]. We compared *unc-3(lf) ain-1(lf)* to *unc-3(lf) ain-1(lf); daf-16 (mu86 lf)* mutants. We found that a mutation in *daf-16* completely suppressed the dauer formation of *unc-3(lf) ain-1(lf)* mutants at 25°C (Figure 1D). Taken together, these results suggest that the *unc-3(lf) ain-1(lf)* animals may disrupt functions that promote the activity of the TGF-β and IIS pathways.

### Major miRNA roles in dauer formation are independent of serotonin activity

**Figure 2:**
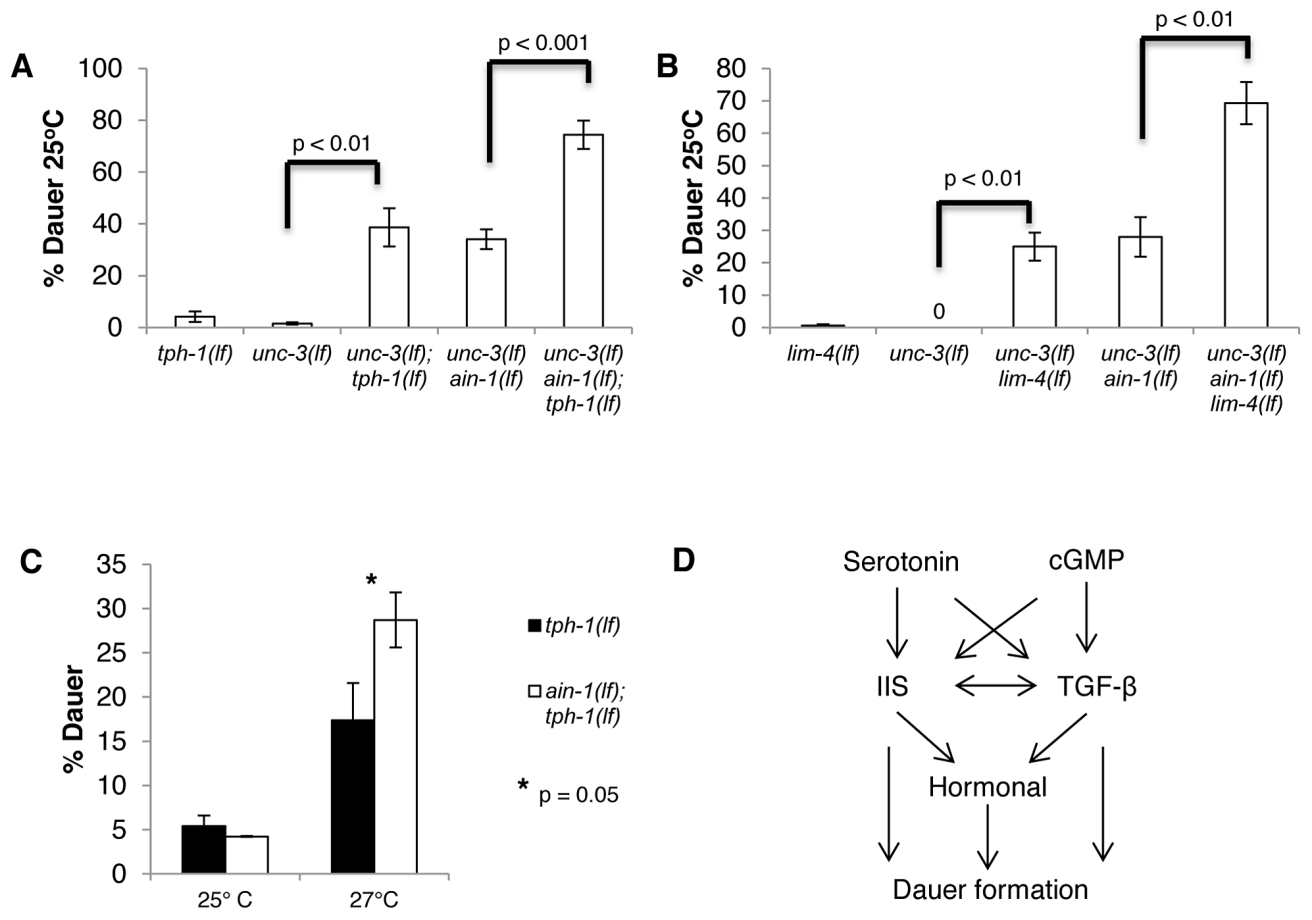
Major neuronal miRNA functions in repressing dauer formation are independent of serotonin activity. **A–C.** Bar graphs representing the percent of dauer progeny of indicated genotypes. **A.** All mutants were assayed in the same experiments. **B.** *unc-3(e151 lf) ain-1(ku322 lf)* and *unc-3(e151 lf) ain-1(ku322 lf) lim-4(yz12 lf)* were scored in separate assays from the *unc-3(lf) lim-4(lf)* mutant. **C.** Original *tph-1(mg280 lf)* mutants were used in this experiment (not siblings of *ain-1(lf); tph-1(lf)* mutants). Error bars represent S.E.M. from at least three biological replicates. A student's t-test was used to determine statistical significance for all experiments. **D.** Brief pathway outlining the relationship of serotonin, cGMP, IIS, and TGF-β in relation to dauer formation.

The observation that miRNAs were required in neurons to repress aberrant dauer formation, and the suppressions of the *unc-3(lf) ain-1(lf)* double mutant phenotype by hyperactive TGF-β and IIS signaling, raised the possibility of defects upstream of both TGF-β and IIS. Serotonin has been suggested to regulate both TGF-β and IIS signaling in *C. elegans* (Figure 2D) [19], [20]. Additionally, a previous study has shown that compromising serotonin signaling, by mutations in the serotonin biosynthesis gene *tph-1* (tryptophan hydroxylase), could enhance the dauer formation of *unc-3(lf)* mutants (Figure 2A) [21]. Consistent with this observation, we found that mutations in the LIM-4 homeodomain protein, which decreases TPH-1 expression in ADF neurons, also enhanced the dauer formation of *unc-3(lf)* mutants (Figure 2B) [22]. Combined, these results suggest that serotonin, produced through TPH-1 in ADF neurons, is important in preventing dauer formation in an *unc-3(lf)* mutant. Interestingly, the rate of dauer formation of the *unc-3(lf); tph-1(mg280 lf)* mutant and the *unc-3(lf) lim-4(yz12 lf)* mutant was similar to that of the *unc-3(lf) ain-1(lf)* mutant (Figure 2A–2B).

To test whether miRNAs regulate serotonin, we compromised serotonin synthesis in the *unc-3(lf) ain-1(lf)* mutant. We found that both the *tph-1(lf); unc-3(lf) ain-1(lf)* and *lim-4(lf) unc-3(lf) ain-1(lf)* triple mutants had significant increases in dauer formation rate when compared to the corresponding double mutant (Figure 2A–2B). Thus, regulation of dauer formation by miRNAs is largely independent of serotonin production, and the Daf-c phenotype associated with *unc-3(lf) ain-1(lf)* mutants is likely to be unrelated to serotonin activity.

We also compared the rate of dauer formation of *ain-1(lf); tph-1(lf)* mutants with that of *tph-1(lf)* mutants, and compared *lim-4(lf) ain-1(lf)* mutants with that of *lim-4(lf)* mutants. Interestingly, we found that *ain-1(lf); tph-1(lf)* double mutants do not have a higher rate of dauer formation than *tph-1(lf)* at 25°C (Figure 2C). Rather, the enhancement of dauer formation is evident at 27°C, confirming that at least a portion of miRNA activity functions in parallel to serotonin to regulate dauer formation (Figure 2C). These results also suggest that compromising both *ain-1* and *tph-1* is not sufficient to generate the strong dauer phenotype. Instead, an additional insult is necessary for the dramatic induction of dauer formation. Thus, the presence of another condition (*unc-3* mutant background or 27°C) reveals the effect of miRNA and serotonin activity on regulating dauer formation. Strangely, *lim-4(lf) ain-1(lf)* mutants and *lim-4(lf)* mutants do not show any increase in the rate of dauer formation, even at 27°C (Figure S1D). This suggests that the *lim-4(lf)* and *tph-1(lf)* mutants differ in their response to a 27°C environment, or a potential difference in the genetic backgrounds in the *lim-4(lf)* and *tph-1(lf)* mutant strains.

### miRNAs may modulate cGMP signaling

**Figure 3:**
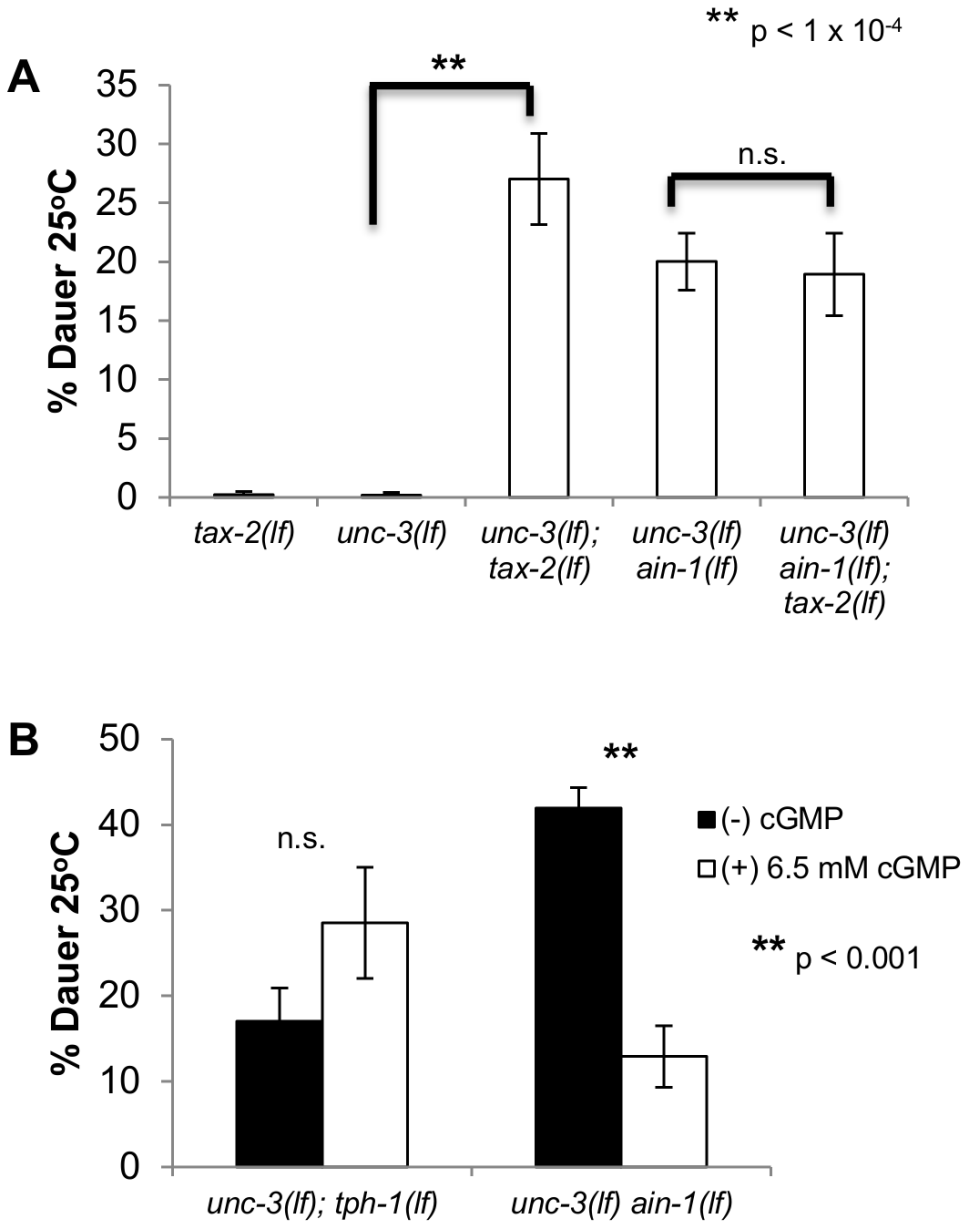
miRNAs may modulate cGMP signaling. **A and B.** Bar graphs representing the percent of dauer progeny of indicated genotypes and conditions. **A.** All mutants were assayed in the same experiment. Alleles used are *unc-3(e151 lf), ain-1(ku322), and tax-2(p691 lf)* **B.** The original *unc-3(e151 lf) ain-1(ku322 lf)* mutant was used in this experiment. This assay was scored blindly between (−)/(+) cGMP plates. Each trial consisted of four independent plates, and the median percent dauer between scored plates was taken for each trial. Error bars represent S.E.M. from at least three biological replicates. A student's t-test was done to determine statistical significance for all experiments.

Similarly to serotonin, cGMP influences both TGF-β and IIS activities as evidenced by changes in TGF-β/DAF-7::GFP and insulin/DAF-28::GFP expression in mutants that lack DAF-11/guanylate cyclase [23], [24]. To test whether cGMP signaling was altered, we compromised cGMP signaling by introducing a mutation into the TAX-2/4 cGMP-gated channel in *unc-3(lf)* and *unc-3(lf) ain-1(lf)* mutants. We found that *unc-3(lf); tax-2(p691 lf)* mutants formed constitutive dauers at a higher rate than *unc-3(lf)* mutants. Moreover, there were no differences between *unc-3(lf) ain-1(lf)* and *unc-3(lf) ain-1(lf); tax-2(lf)* mutants (Figure 3A). These observations suggest that defects in cGMP signaling, in combination with the *unc-3(lf)* mutant background, can cause constitutive dauer formation. This is consistent with the observation that compromised cGMP signaling can synergize with mutations in TGF-β mutants because *unc-3(lf)* have compromised TGF-β signaling [7], [25]. More interestingly, these results suggest that cGMP signaling is altered in *unc-3(lf) ain-1(lf)* mutants and that neuronal miRNAs may modulate cGMP signaling.

To investigate if miRNAs affected the levels of cGMP or the cGMP signal transduction machinery, we exogenously supplemented *unc-3(lf) ain-1(lf)* mutants with the 8-bromo-cGMP analog, which has been shown to decrease dauer formation of *daf-11(lf)* mutants but not TGF-β mutants [26]. By increasing the levels of cGMP, we were able to decrease the rate of dauer formation of *unc-3(lf) ain-1(lf)* mutants (Figure 3B). Additionally, we saw that increasing the levels of cGMP failed to decrease the rate of dauer formation of *unc-3(lf) tph-1(lf)* mutants, suggesting that the effect of 8-bromo-cGMP supplementation is specific to the dauer formation of *unc-3(lf) ain-1(lf)* and not general *unc-3(lf)*-involved dauer formation. Interestingly, the supplementation of 8-bromo-cGMP to *unc-3(lf) tph-1(lf)* mutants seems to cause an increased rate of aberrant dauer formation, the reason for this is unclear. Because we can partially rescue the Daf-c phenotype by increasing levels of cGMP, it is likely that a portion of miRNA activity is dedicated to maintaining cGMP signaling, either directly or indirectly. However, the inability to completely suppress dauer formation through 8-bromo-cGMP supplementation suggests that miRNAs are not only influencing cGMP signaling, but are also likely regulating other mechanisms of dauer signaling.

Overall, these results suggest that dauer formation of *unc-3(lf) ain-1(lf)* is caused, in part, by defects in maintaining proper cGMP signaling. Moreover, the evidence that *ain-1(lf)* mutant animals do not form constitutive dauers similar to *daf-11(lf)* mutants suggests that cGMP signaling is only modestly affected by miRNAs. Interestingly, the modest regulation of cGMP signaling by miRNAs is only revealed in the *unc-3(lf)* mutant background, as defects in cGMP-dependent dauer formation are only apparent in the *unc-3(lf) ain-1(lf)* mutant and not the *ain-1(lf)* mutant.

### Multiple neuronal miRNAs regulate dauer formation

Our genetic evidence for a function of neuronal miRNAs in regulating dauer formation prompted us to further analyze neuronal miRNAs by a biochemical approach. We applied tissue-restricted immunoprecipitations of neuronal miRISCs from an asynchronous population of worms, as we have done previously for the intestine and muscle [4]. Briefly, a neuronal specific rgef-1^prom^::AIN-2::GFP transgene, which can rescue the *unc-3(lf) ain-1(lf)* phenotype (Figure S1B), was integrated into the genome through single-copy ballistic transformation. An antibody against GFP was used for immunoprecipitation to enrich for neuronal miRISCs.

**Figure 4:**
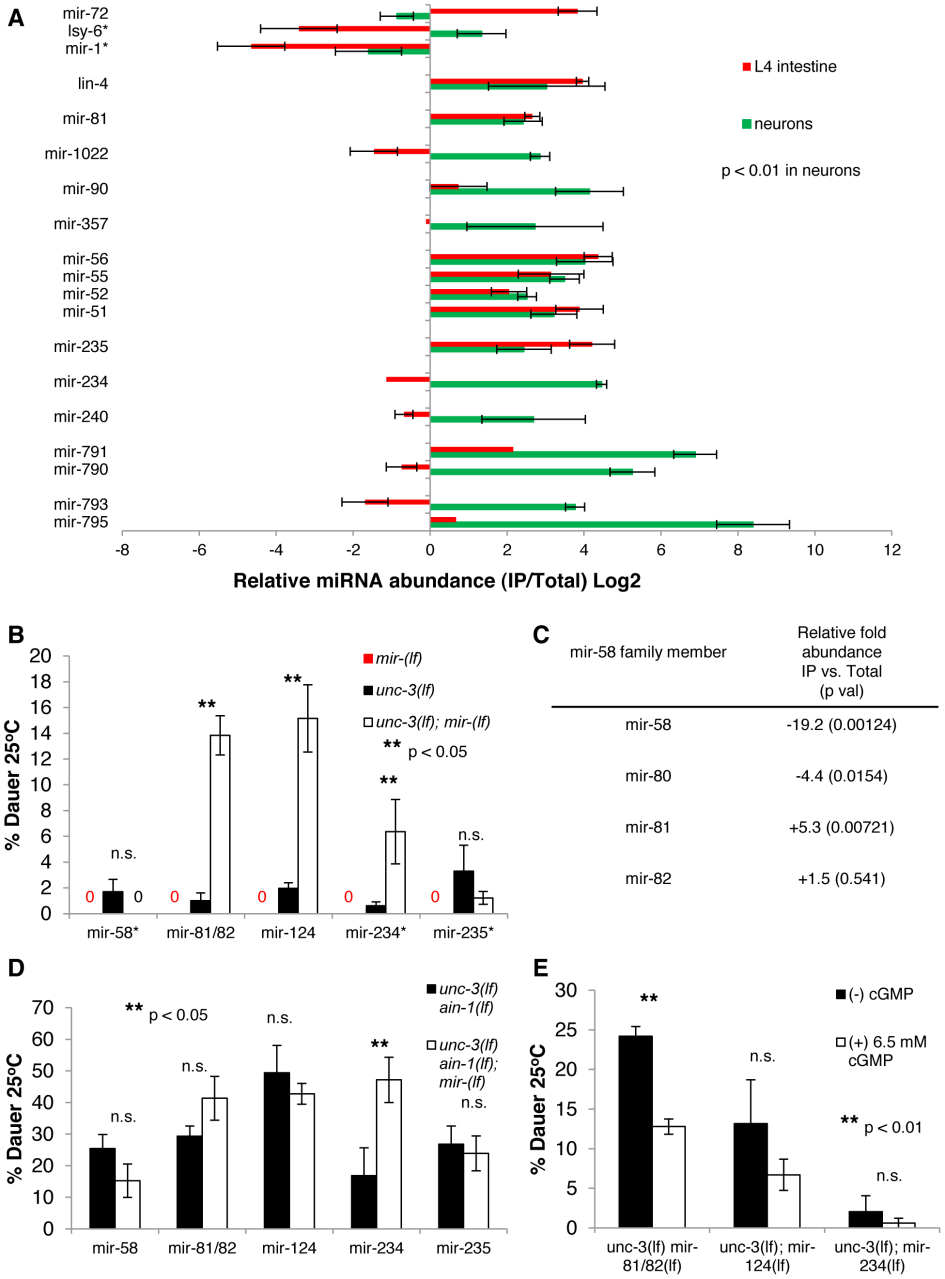
Multiple neuronal miRNAs modulate dauer signaling. **A.** Bar graph representing the relative abundance of miRNAs enriched in the asynchronous neuronal miRISC IP, with a p<0.01 determined by a student's t-test (* except miR-1 and lsy-6). Error bars represent S.E.M. from four biological replicates. **B.** Bar graph representing the percent of dauer progeny of indicated genotypes. Assays for each *unc-3(lf)* and *unc-3(lf); miR(lf)* pair was done in parallel, but independently from other miRNA mutants (i.e. *unc-3(lf)* and *unc-3(lf); miR-124(lf)* were run in parallel to each other, but not in parallel to *unc-3(lf) miR-81/82(lf)* mutants). The original *unc-3(lf)* was used compared to *unc-3(lf); miR-124(lf)*. * indicates that the individual miRNA mutant was run separately from the *unc-3(lf)* and *unc-3(lf); miR-(lf)* mutant. Error bars represent S.E.M. from at least three biological replicates. A student's t-test was used to determine statistical significance between *unc-3(lf)* and *unc-3(lf) miR-(lf)*. Alleles used are *unc-3(e151 lf)*, *ain-1(ku322 lf)*, *miR-58(n4640 lf)*, *miR-81/82(nDf54 lf)*, *miR-124(n4255 lf)*, *miR-234(n4520 lf)*, and *miR-235(n4504 lf)*. **C.** Chart showing the relative abundance (IP vs. Total) of miR-58 family members and statistical significance determined by a student's t-test from deep-sequencing experiments. **D.** Bar graph representing the percent of dauer progeny. Assays for each miRNA mutant was run in parallel to the corresponding *unc-3(lf) ain-1(lf)* mutant but not in parallel to assays for other miRNA mutants (which may have resulted in the variation seen in *unc-3 (lf); ain-1(lf)* mutants across different experiments). The original *unc-3(lf) ain-1(lf)* strain was used as a control in miR-81/82 and miR-124 experiments. Error bars represent S.E.M from at least three biological replicates. **E.** Bar graph showing the percent of dauer progeny. Assays were scored blindly between (−)/(+) cGMP. Error bars represent S.E.M. from at least three biological replicates and a student's t-test was used to determine statistical significance.

We subjected four biological replicates of an asynchronous population to immunoprecipitation, small RNA isolation, and Illumina deep-sequencing. We identified 16 miRNAs that were significantly enriched in neurons when compared to total whole worm lysate (p<0.01) (Dataset S1). Of the 16 miRNAs we identified, 12 of these have been analyzed by promoter::GFP analysis [27]. Of these 12 miRNA promoters, 9 show some indication of having neuronal expression (Dataset S1). Despite the previous lack of visible GFP expression of the other 3 miRNAs in neurons, this method was sensitive enough to detect the enrichment of the mature miRNA sequence within neurons. Additionally, our neuronal miRISC IP identified 33 miRNAs that were highly depleted from neurons when compared to total whole worm lysate. Of these 33 depleted miRNAs, 18 were analyzed by promoter::GFP analysis, and 12 showed no indication of being expressed in neurons. Thus a portion of the miRNAs identified in our IPs is supported by previous promoter::GFP analysis. Moreover, the subset of miRNAs we identified in this asynchronous, neuronal immunoprecipitation is different from our previously identified L4 intestine-specific miRNAs (Figure 4A). This comparison is limited in the fact that the miRNAs are from a different stage and a different tissue, but the methods used to isolate, clone, and sequence the miRNAs between these two datasets were exactly the same and were the most comparable. However, our data did not show a statistically significant enrichment of lsy-6, a known neuron-specific miRNA (p<0.15). Because lsy-6 is expressed in a small subset of chemosensory neurons [28], and our systematic investigation of neuronal miRISCs did not discriminate between neuronal subtypes, this method may not be sensitive enough identify miRNAs that are expressed in a small subset of neurons.

The neuronal specificity of this IP analysis is also consistent with the following findings. First, we noticed a moderate depletion of miR-1, a muscle-specific miRNA, from neuronal miRISCs (p<0.06) [4], [29]. Second, this neuronal miRISC IP was depleted for miRNAs that were enriched in the intestine, such as miR-72 [4]. Finally, we observed that several miRNAs enriched in the neuronal IP were depleted from the intestinal IP, such as miR-790. Although there are some limitations, the miRNAs identified in this neuronal IP are a subset of miRNAs that are highly expressed in neurons.

To test the roles of several of these identified miRNAs in dauer formation, we ablated the function of single miRNAs in *unc-3(lf)* mutants. In addition to *mir-81*, *mir-234*, *mir-235*, and *mir-240*, which were identified in our IPs, we also tested *mir-124*, which is a highly conserved neuronal miRNA that was not enriched in the IP [30]. We found that deletion mutations in *mir-81/82*, *mir-234*, or *mir-124* significantly enhanced the Daf-c phenotype of *unc-3(lf)* mutants (Figure 4B and Figure S1E), suggesting that these miRNAs have a role in dauer formation. Because the rate of dauer formation of each of these double mutants was less than that of the *unc-3(lf) ain-1(lf)* double mutant, these results suggest that there is a complex network of regulation exerted by multiple miRNAs in modulating the neuronal component of dauer formation. Additionally, we found that ablation of *mir-81/82*, *mir-124*, or *mir-235* in *unc-3(lf) ain-1(lf)* did not further increase the rate of dauer formation (Figure 4D). This observation suggests that the *ain-1(lf)* background compromises a sufficient amount of activity of these miRNAs to exert a detectable phenotype. However, we found that further deletion of *mir-234* in the *unc-3(lf) ain-1(lf)* mutant increased the rate of dauer formation, relative to its double mutant sibling control. However, the absolute rate of dauer formation of the *unc-3(lf) ain-1(lf); mir-234(lf)* mutant is no different than the other *unc-3(lf) ain-1(lf)* clones (i.e. the *unc-3(lf) ain-1(lf)* control for *mir-81/82 or mir-124*), which may simply be a result of a varying genetic background introduced specifically in the *unc-3(lf) ain-1(lf)* control clone for *mir-234*. Nonetheless, the finding that these specific miRNAs function in repressing aberrant dauer formation in an *unc-3(lf)* mutant background supports the validity of this tissue-specific-IP approach in identifying neuronal miRNAs.

Because of the effect of *mir-81/82* in repressing aberrant dauer formation in an *unc-3(lf)* mutant background, we tested *mir-58*, a family member of *mir-81/82*. We found that *unc-3(lf); mir-58(lf)* mutants behaved similarly to the *unc-3(lf)* mutant and that *unc-3(lf) ain-1(lf); mir-58(lf)* mutants were similar to *unc-3(lf) ain-1(lf)* mutants in dauer formation (Figure 4B, 4D). A previous study found that the mir-58 family is required for dauer formation, as deletion of the entire family (*mir-58, -80, -81, -82*) resulted in an inability to form dauer larvae (Daf-d) [2], which is opposed to the function of the miRNAs discussed above. Interestingly, they found that restoring *mir-81* in this familial deletion did not rescue the Daf-d phenotype, which is consistent with our observation of *mir-81* repressing dauer formation. Moreover, we found *mir-58* to be 19-fold depleted from neuronal miRISC (p = 0.00124) while *mir-81* was enriched in neuronal miRISC (Figure 4C). Thus, although family members can function redundantly in targeting similar mRNAs, the spatial expression of different miRNA family members can allow for different, even opposing, biological functions.

We also tested cGMP supplementation on *unc-3(lf); mir-81/82(lf)*, *unc-3(lf); mir-124(lf)*, and *unc-3(lf); mir-234(lf)* mutants. We found that cGMP could partially decrease the rate of dauer formation of *unc-3(lf); mir-81/82(lf)* mutants but had a variable effect on the other two mutants (Figure 4E). These data suggest that different miRNAs modulate different aspects of dauer signaling and that *mir-81/82* has a potential role in cGMP signaling. Additionally, we asked whether the *unc-3(lf) mir-81/82(lf); mir-124(lf)* triple mutant had an increased rate of dauer formation when compared to either the *unc-3(lf) mir-81/81(lf)* or the *unc-3(lf); mir-124(lf)* mutants. Interestingly, we found no robust difference in the rate of dauer formation between the *unc-3(lf) mir-81/82(lf)* and the *unc-3(lf) mir-81/82(lf); mir-124(lf)* mutant (Figure S1F). This may suggest that even though these two miRNAs can modulate different aspects of dauer signaling, their overall effects on dauer signaling may be similar. For example, if miR-81 influences cGMP signaling, which indirectly influences IIS and TGF-β signaling, other miRNA influences on these pathways may be limited.

### Identification of neuronal miRNA targets

**Figure 5:**
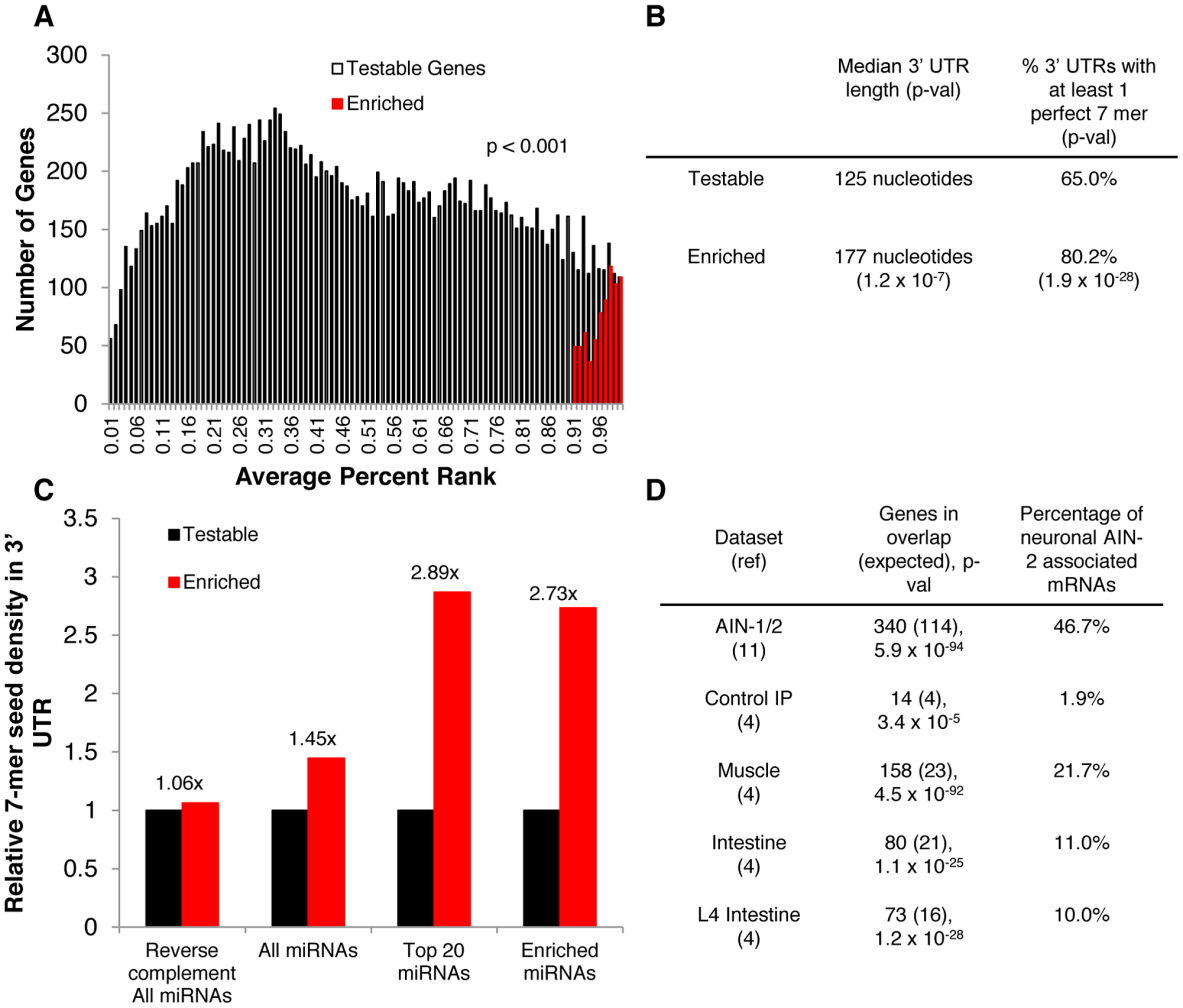
mRNAs associated with neuronal miRISC are likely neuronal miRNA targets. **A.** Bar graph showing the distribution of genes identified through microarrays analysis. Testable genes were defined as having reliable signals in at least two microarray experiments (see Methods). For multiple probes for a single transcript, the probe with the smallest p-value from a one-tailed t-test was used (see Methods). For alternative transcripts to a single gene, the transcript with the smallest p-val was used. There were 17673 testable genes and 747 enriched genes in the graph. **B.** Chart showing the median length of 3′ UTRs and the percentage of 3′ UTRs with at least one perfect 7-mer binding site to all annotated *C. elegans* miRNAs. A nonparametric median test was used to determine the difference between median 3′ UTR length (see Methods). Statistics from a hypergeometric distribution was used for determining the difference in percentages of having at least one 7-mer. **C.** Bar graph that shows relative seed density. Seed density is calculated by (# of perfect 7-mer seed matches/1000 nt UTR). The density is relative to that of testable genes. Enrichment values shown above enriched genes (i.e. 1.45×) show relative changes in seed density compared to control. “Top 20 miRNAs” indicates the miRNAs, with the top 20 highest raw read counts in our IPs. “Enriched miRNAs” indicates miRNAs that were enriched and statistically significant in our IPs. **D.** Chart showing the overlap between genes identified in previous datasets to genes associated with neuronal AIN-2. P-values and expected values were calculated using a hypergeometric distribution. Percentages represent 100*[(# genes in overlap)/(# genes associated with neuronal AIN-2)].

In addition to identifying neuronal miRNAs, we applied microarray analysis to identify potential miRNA targets that co-immunoprecipitated with asynchronous neuronal miRISCs, in five biological replicates (Dataset S2). In each replicate, the ratio of IP signal/Total signal was converted to percent ranks (Figure 5A), as described previously [4], [31]. Through our microarray analysis, we identified 747 (p<0.001) probes that mapped to unique genes and that were associated with neuronal miRISCs. Of these 747 probes, 728 were readily identified with a gene. In order to confirm whether these associated mRNAs were miRNA targets, we analyzed the 3′ UTRs of these associated genes. We found that the 3′ UTRs of these genes were enriched for perfect 7-mer (2–8 nt) binding sites of *C. elegans* miRNAs per 1000 nucleotides (Figure 5C). In contrast, there was minimal enrichment when the reverse complement of perfect 7-mer binding sites was used in the analysis. Moreover, the relative enrichment of perfect 7-mer binding sites was even more robust for miRNAs that we identified through our neuronal IP and deep-sequencing analysis. Additionally, the median 3′ UTR length of the subset of these mRNAs is longer than the median 3′ UTR of all testable genes in our microarray experiments (Figure 5B). We also found that the percentage of genes with at least one perfect 7-mer binding site (to all *C. elegans* miRNAs) was higher for genes associated with neuronal miRISCs than for all testable genes. These observations are similar to what was observed in our previous intestine- or muscle-restricted IPs [4]. Furthermore, we compared our data against our previous IPs and found that a portion of genes enriched in neuronal miRISC were also enriched in our whole body, muscle, and intestine IPs, suggesting that we had previously identified a subset of these miRNA targets (Figure 5D). Collectively, these observations suggest that the genes identified through our IPs are likely miRNA targets in neurons.

Additionally, we compared the relative mRNA levels of several of our top targets in *ain-1(ku322 lf); ain-2(tm1863 rf)* double mutants and N2 worms, as done previously [4] (Figure S2). We found that 8/9 genes that we tested show a higher relative mRNA level in *ain-1(lf); ain-2(rf)* worms than N2. The last gene that did not show a higher relative mRNA (*lev-1*), was shown previously to be up-regulated by a GFP 3′ UTR analysis, suggesting that regulation may occur via translational efficiency. It is important to note, however, that the up-regulation of these 8 genes may be indirect, but their association with neuronal miRISC supports the idea that their up-regulation is likely a result of lack of direct miRNA targeting. Overall, these results support the idea that the mRNAs associated with neuronal miRISC are up-regulated when miRISC is compromised.

**Figure 6:**
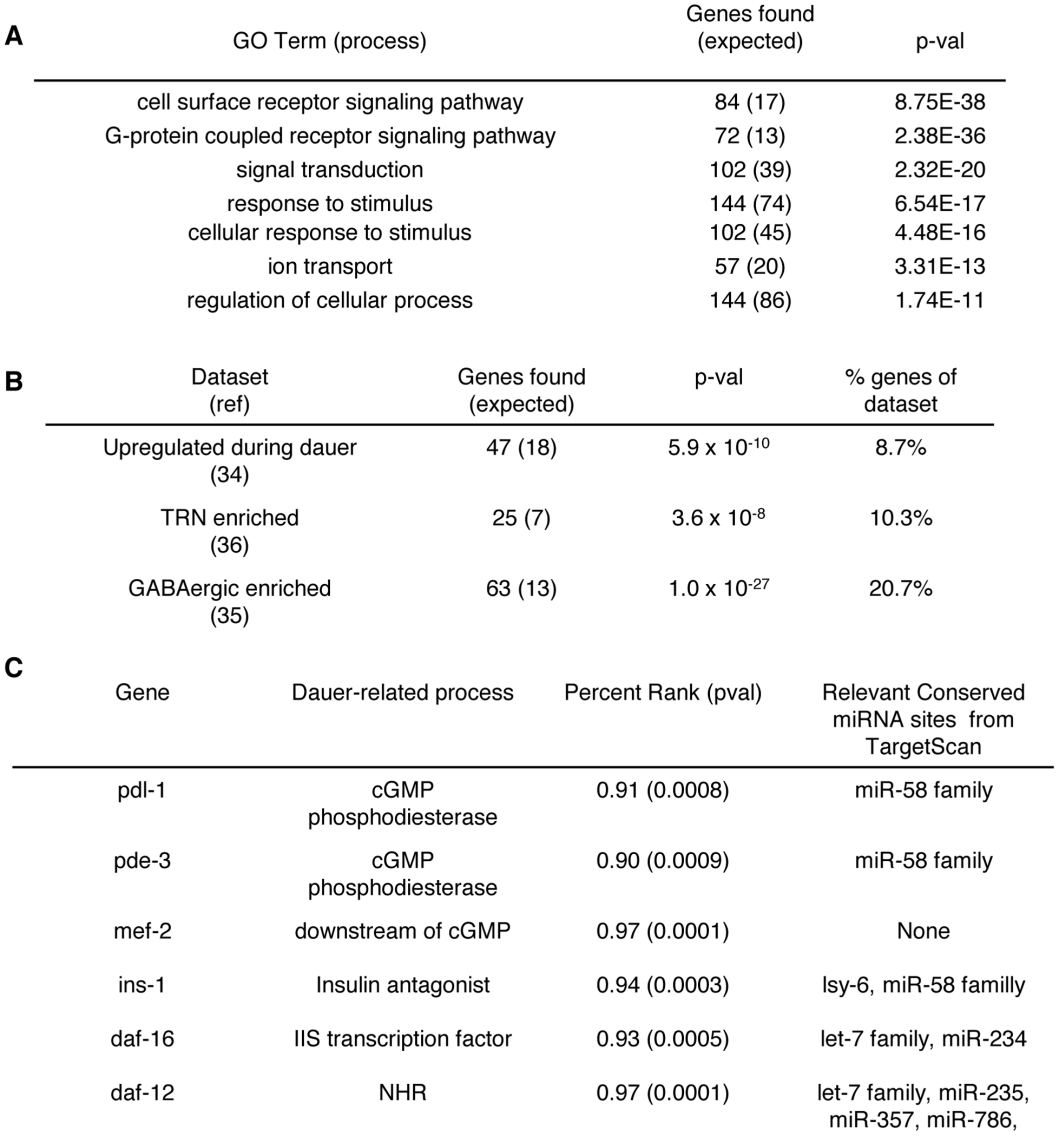
Neuronal miRNA targets have broad functions. **A.** Chart showing the top GO term enrichments of enriched genes versus testable genes using GOrilla. 9873 testable genes and 531 enriched genes were identified with GO terms. P-values and expected values were calculated using a hypergeometric distribution. **B.** Chart showing the enrichment of genes from various datasets within our neuronal AIN-2 IP. P-values and expected values were calculated using a hypergeometric distribution. **C.** Chart showing several genes that promote dauer formation and are associated with neuronal AIN-2. Relevant Conserved sites are sites that have some indication of belonging to a neuronal miRNA in this study or others.

To further characterize the tissue-specificity of our IP, we compared the average enrichment of genes that were known to be expressed in various tissues through previous IP-microarray analysis of PAB-1 associated mRNAs in specific tissues [12], [31], [32]. The average percent rank of all testable genes in our dataset was 0.48. We found that the average percent rank of genes highly expressed in muscle and intestine to be 0.51 (p = 1.4×10^−4^) and 0.45 (p = 1.66×10^−5^), respectively (Figure 6A) [31], [32]. Thus, there is a small enrichment of highly expressed muscle genes in our dataset. In contrast, genes that are highly expressed in the intestine are depleted from the neuronal miRISC IP. Not surprisingly, we found minimal overlap between genes enriched in our neuronal miRISC IP with genes highly expressed in either muscle or intestine. We found that the average percent rank of transcripts that are highly expressed in neurons to be 0.73 (p = 5.5×10^−241^), with extensive overlap between genes enriched in the neuronal miRISC IP and genes highly expressed in neurons. Moreover, we found that genes that were highly expressed in neurons, but not associated with neuronal miRISCs, also had a percent rank higher than average (data not shown). Thus, the overlap between neuronal miRISC-associated genes and highly expressed neuronal genes is not responsible for the enrichment of neuronal genes in our dataset. Overall, these data suggest that our neuronal IP is specific for neuronal genes.

The enrichment of highly expressed neuronal genes in our dataset could suggest that we were simply systematically enriching for genes that were expressed at higher levels in neurons. However, the possibility that the AIN-IP method favors genes expressed at higher levels was dismissed in our previous analysis of intestine- and muscle-restricted IPs [4]. To test if this was also true in neurons, we further analyzed the 3′ UTRs of the neuronal and intestinal PAB-1 datasets [11], [32]. We saw a similar trend in the neuronal PAB-1 dataset as we did with our neuronal miRISC dataset. As a reference, we used all 3′ UTRs identified previously [33]. Genes that are highly expressed in neurons have a longer median 3′ UTR, more perfect 7-mer seed sites within the 3′ UTR, and a higher percentage of genes with at least one perfect 7-mer than all 3′ UTRs in the database (Figure S3). This contrasts with genes that are highly expressed in the intestine. Additionally, these neuronally enriched genes are also slightly enriched in our previous intestine- and muscle-restricted IPs (Figure S3). Overall, these data suggest that a proportion of neuronally expressed genes are miRNA targets. Thus, the enrichment of previously identified neuronal genes in our neuronal miRISC IP indicates that the previous neuronal PAB-1 IP is also enriched for miRNA targets. Interestingly, this may suggest that genes highly expressed in neurons are more likely to be regulated by miRNAs than genes in other tissues.

### Neuronal miRNA targets have broad functions

We analyzed mRNAs that were significantly associated with neuronal miRISCs by utilizing GOrilla (http://cbl-gorilla.cs.technion.ac.il/), an online resource for GO term enrichment analyses (Table S1). We found that a wide range of GO terms involved with neuronal activity were enriched in neuronal miRISC associated genes when compared to testable genes (Figure 6A). These GO term enrichments show that a wide variety of processes may be modulated by neuronal miRNAs. Additionally, we looked at previous genes that were enriched in dauer larvae, in GABA neurons, and in touch receptor neurons [34], [35], [36]. We found that genes from these datasets were overrepresented within the subset of genes associated with neuronal AIN-2 (Figure 6B). This overrepresentation may imply that neuronal miRNAs help regulate these processes, reinforcing the idea the neuronal miRNAs have broad physiological functions.

We searched for genes that promote dauer formation within our dataset and found a variety of genes that affected multiple aspects of dauer signaling and looked at their 3′ UTRs for potential miRNA binding sites via TargetScan (Figure 6C) [33]. For example, *pde-3* and *pdl-1* were enriched in our IPs. Both genes encode for predicted cGMP phosphodiesterases that may regulate cGMP levels. Also, *mef-2* which functions downstream of EGL-4 and cGMP signaling, was also enriched in our IPs [37]. Thus, aspects of cGMP are likely regulated by miRNAs. Additionally, *ins-1*, an insulin antagonist, and *daf-16* were enriched in our IPs, indicating possible miRNA modulation of insulin signaling. *daf-12*, a nuclear hormone receptor, was also identified in our IPs, which has been previously shown to be regulated by miRNAs to influence dauer formation [38]. In addition to identifying genes that promote dauer formation, we found several genes that repress dauer formation (*daf-4*, *daf-28*, insulin-like-peptides). However, it is unclear whether or not there is a switch between targeting genes that promote dauer formation versus genes that repress dauer formation in response to specific environmental stimuli. The identification of many dauer-signaling related mRNAs and genes enriched in dauer larvae suggests that miRNA regulation over dauer formation is complex. It is likely that multiple miRNAs target multiple genes that control several different dauer pathways. Moreover, the broad function of miRNAs in modulating neuronal activity may also influence dauer signaling.

## Discussion

Understanding the role and importance of miRNA regulation on physiological processes is a challenging aspect of miRNA biology that has often proven to be difficult using classical genetics. This difficulty is likely due mainly to two important aspects of miRNA-involved regulation of gene expression. First, miRNAs often function to enhance the robustness of gene expression related to specific physiological functions; disrupting miRNA regulation may not alter expression to the extent that generates phenotypes observed by established assays. Second, miRNA roles in a specific physiological process are often executed as a miRNA-target interaction network involving multiple miRNAs and a large number of their targets [1], [2], [4], [5], [39], [40]. Therefore, in this study, we used a sensitized genetic background to interrogate miRNA functions, similarly to previous studies [3], [4], [5]. Within the *ain-1(lf)* genetic background, we found that compromising UNC-3 resulted in an enhancement of the *unc-3(lf)* Daf-c phenotype. Thus, the *unc-3(lf)* genetic background permitted the discovery of functions of miRNAs that would have otherwise been masked by “genetic redundancy” [5]. The induction of dauer formation is a physiological process that orchestrates organismal-level changes. As such, the process is tightly regulated through a variety of different mechanisms involving cGMP, serotonin, TGF-β, IIS, and hormonal signaling between various tissues [15]. Few miRNAs have been functionally linked to dauer formation, except for the *miR-58* and *let-7* family of miRNAs [2], [38].

However, through our genetic investigations of the *unc-3(lf) ain-1(lf)* mutant, we have shown a physiological role of neuronal miRNAs in repressing aberrant dauer formation, through possible modulation of cGMP signaling and other unidentified mechanisms. Moreover, we have biochemically identified miRNAs that are highly expressed in neurons and verified the function of several of these individual miRNAs in dauer formation. Again, the use of the *unc-3(lf)* mutant background has allowed us to discover physiologically important functions of these individual miRNAs. For example, a previous study on miR-124 observed no overt phenotypes for the *mir-124(lf)* mutant [30]. But, a physiological function of miR-124 is revealed within the *unc-3(lf)* mutant background. Thus, within various genetic backgrounds, the biological function of miRNAs can be further elucidated.

Our investigation into functions of neuronal miRNAs highlights the complexity behind miRNA regulation and genetic redundancy. We identified a function of several individual miRNAs in modulating dauer formation, thus revealing that multiple miRNAs can function similarly in modulating the same biological process. Because these miRNAs have different seed sites, it is likely that they target different subsets of mRNAs, which may or may not overlap. Moreover, it is likely that the collective misregulation of many miRNA target genes, in combination with the genes misregulated under the *unc-3(lf)*, interact to result in an overt phenotype, and dissecting specific miRNA::miRNA target interactions will prove difficult.

Furthermore, even though we observed that the collective function of miRNAs is important in modulating dauer formation, we also found that this function depends on the spatial expression of miRNAs. We observed that miRNAs could either function neuronally to repress dauer formation, or partially in the intestine to promote dauer formation. Combined with the observation of a previous study, our data suggests that the differing spatial expression of *miR-58* family members may allow for opposing biological functions, adding to the complexity of the system [2]. Thus, analyzing the function of miRNAs in a specific tissue can allow for a clearer understanding of their impact on cellular and organismal physiology as exemplified previously [4].

By identifying miRNAs and potential mRNA targets within neuronal tissue through our systematic co-immunoprecipitation analyses, we discovered that neuronal miRNAs target mRNAs that are involved in many different neuronal activities. As an example, we found that genes that are enriched in dauer larvae were overrepresented in our neuronal miRISC IP. Thus, it may be plausible that miRNAs serve as a parallel mechanism to repress these genes, independent of dauer signaling pathways, under non-dauer inducing conditions. However, we also found that miRNAs also target mRNAs involved in known dauer signaling pathways, possibly reinforcing the robustness of these mechanisms. Interestingly, targeted mRNAs both promote and repress dauer formation. The precise mechanism by which mRNAs that promote and repress dauer formation differentially associate with neuronal miRISCs under different environmental conditions is intriguing but remains unclear. However, studies investigating changes in miRNA expression in response to dauer formation may provide some insight into this phenomenon [41], [42]. Interestingly, these studies identify changes in *mir-234* in response to stress, and we identified a potential function of *mir-234* in repressing dauer formation in this study.

In addition to mRNAs that were involved with dauer formation, we also found mRNAs that are enriched in GABAergic and touch receptor neurons. However, this systematic approach is unable to distinguish whether these GABAergic mRNAs are expressed in GABA neurons to maintain function, or in other neurons to repress GABAergic qualities in non-GABA neurons. The same thinking applies to the touch receptor neuron genes and further investigation into these processes can be explored. Aside from these processes, we unexpectedly found that neuronal mRNAs seem to have a higher propensity for being regulated by miRNAs than mRNAs in the intestine in *C. elegans*. We believe that the constant role of neurons to relay messages throughout the organism requires tight regulation at multiple levels of neuronal processes to ensure the integrity of neuronal signaling. This is, in part, exerted by miRNAs. The observation that many GO terms involved in neuronal activity were enriched in our neuronal miRISC IP reveals the broad functions of neuronal miRNAs and supports the idea of neuronal miRNAs maintaining neuronal activity. Additionally, it may be possible that miRNA regulation may be well-suited for regulating neuronal genes that require translation along the axon.

Overall, our investigation of miRNA function in neurons emphasizes the complexity of miRNA function. By identifying hundreds of associated mRNAs with neuronal miRISCs, we have only begun to understand the physiological role of these mRNAs. We have already identified a functional role of miRNAs in modulating dauer formation, but the specific miRNA::miRNA target interactions responsible for this process will be difficult to dissect. Like dauer formation, the other function of neuronal miRNAs may be masked by other neuronal controls of gene expression and using sensitized backgrounds, which compromise these other controls of gene expression, may help uncover other important roles of neuronal miRNA regulation.

## Materials and Methods

### Worm methods

Worms were maintained at 20°C on OP50 *E. coli* unless otherwise noted. The wild-type strain was Bristol strain, N2. Mutant alleles used were *ain-1(ku322)X, ain-1(tm3681)X, unc-3(e151)X, ain-2(tm2432)I, ain-2(tm1863)I, unc-31(e928)IV, daf-5(e1386)II, daf-16(mu86)I, tph-1(mg280)II, lim-4(yz12)X, tax-2(p691)I, unc-119(ed3)III (for single-copy integration), miR-58(n4640)IV, miR-81/82(nDf54)X, miR-124(n4255)IV, miR-234(n4520)II, and miR-235(n4504)I*.

### Generation of transgenic lines

The transgenic strains used to perform immunoprecipitations were constructed as previously described, with the addition of a 3 kb region upstream of *rgef-1* used as a pan-neuronal promoter [4], [10]. For transgenic animals used in dauer assays, AIN-1 was translationally fused to GFP into PBSIIKS. The 3.1 kb region upstream of *ain-1* was used as the endogenous promoter, a 3 kb region upstream of *rgef-1* was used as a neuronal promoter, and a 3.1 kb region upstream of *ges-1* was used as an intestinal promoter. A fosmid spanning *unc-3* was used for transgenic rescue (WRM0622bH08). Transgenic animals used in dauer assays were made through microinjections.

### Dauer assays

L4/Young adults grown at 20°C were singled onto OP50 plates and placed at 25°C. Progeny were scored 65–90 hours after worms were picked. Each trial consisted of three plates. At least three biological replicates were completed for each strain unless otherwise noted. The total percent of dauer progeny, from the three plates, was counted and larvae younger than L2 were ignored. For transgenic animals, L4/young adults containing the transgene of interest were singled. Scoring distinguished between transgenic and non-transgenic siblings within the same plate. The percent of dauer formation for both transgenic and non-transgenic animals was normalized to the percent of dauer progeny of non-transgenic animals. Assays comparing miRNA mutants were done by blindly scoring plates of *unc-3(lf)* vs. *unc-3(lf); mir(lf)* and *unc-3(lf) ain-1(lf)* vs *unc-3(lf) ain-1(lf); mir-(lf)*. Additionally, assays involving additional mutations in an *unc-3(lf)* or *unc-3(lf) ain-1(lf)* background were compared to *unc-3(lf)* or *unc-3(lf) ain-1(lf)* clones derived from non-mutant siblings of heterozygous parents, unless otherwise noted. For example, in assays comparing *unc-3(lf)* and *unc-3(lf); mir-58(lf)* mutants, both mutants were derived from the same *unc-3(−/−) mir-58(+/−)* parent to minimize genetic differences. Also, assays comparing specific mutants were run on the same day, unless otherwise noted.

### 8-bromo-cGMP supplementation

The procedure was performed as described previously with the following exceptions [25]. 8-bromo-cGMP was used at a concentration of 6.5 mM and was added to 100 ul of fresh overnight OP50 culture and then spotted onto NGM plates.

### Immunoprecipitation and microarray analysis

The procedure was performed on a mixed-stage population of worms as described previously with the following exceptions [4], [10]. First, four of the five biological replicates were analyzed using an Agilent *C. elegans* microarray chip. Testable genes were defined as having reliable signals in two different biological replicates using the Agilent microarray chip. Multiple probes for the same gene were removed, keeping the probe with the lowest p-value as defined by a one-tailed t-test comparing all testable probes versus probes towards a designated gene. The testable data was then supplemented with the enrichment values (as percent ranks) of a fifth replicate, from another microarray platform (which had a single probe for each gene), and statistical significance was re-calculated. In order to utilize the data from both microarray platforms, we decided that keeping the probe with the lowest p-value would be the best way of compiling the data. The percent ranks were not altered by filtering out these probes.

### MicroRNA cloning and analysis, and 3′ UTR analysis

Small RNA isolation, cloning, and analysis was performed essentially as described previously for the L4-staged immunoprecitations [4]. However, the procedure was performed on a mixed-staged population of worms instead of an L4 synchronized population. For the miRNA analysis, the relative concentration of a miRNA was given by (# reads of miRNA)/(# total reads in experiment). This relative concentration was then converted to relative abundance by normalizing every relative concentration to the lowest relative concentration. The relative abundance was then expressed in log2, which was used to compare in IP RNA vs. Total RNA. A nonparametric median test comparing 3′ UTR length can be found at http://www.fon.hum.uva.nl/Service/Statistics/Median_Test.html. To limit data input, we compared 400 points for each dataset (every 0.25 percentile length).

### Quantitative RT-PCR

The procedure was done on asynchronous, well-fed worms grown on OP50 as described previously [4].

### Accession numbers

The Gene Expression Omnibus accession number for the microarray data reported in this paper is GSE45871.

## Supporting Information

Dataset S1 Neuronal miRISC IP – deep sequencing. Analyzed deep sequencing data from 4 biological replicates. Dataset shows relative enrichment values and p-values from a student's t-test.
(XLSX)

Dataset S2 Neuronal miRISC IP – microarray. Analyzed data from 5 biological replicates on two different microarray platforms. Replicates B–E were run with Agilent chips. Replicate A came from another microarray platform. Enrichment value (IP vs Total) of each gene in each replicate is expressed in terms of percent ranks as described previously [4].
(XLSX)

Figure S1 Additional dauer assays. A, D. Chart showing percentage of dauer of indicated strains, two biological replicates were done. B, C. Relative rate of dauer formation of indicated strains at indicated temperature. Data is the average of at least two independent lines. E, F. Rates of dauer formation of indicated strains.
(PDF)

Figure S2 qPCR of potential miRNA targets. Chart showing the relative log2 enrichment of indicated gene in *ain-1(ku322 lf); ain-2(tm1863 rf)* vs N2 from four biological replicates. *indicates only three biological replicates were done.
(PDF)

Figure S3 Relevant to Figure 5. Neuronal genes are likely miRNA targets. A. Chart showing the overlap and enrichment of highly expressed genes in various tissues within the neuronal miRISC dataset. A student's t-test was used to determine differences in average percent rank against all testable genes (average rank of 0.48). P-values and expected values were calculated using a hypergeometric distribution. Percentages were calculated from 100*[(# genes in overlap)/(# genes associated with neuronal AIN-2)]. B. Chart showing median 3′ UTR length and percent 3′ UTRs with at least one perfect 7-mer binding site to all annotated miRNAs, methods for statistics are similar as before. C. Bar graph showing the relative seed density of perfect 7-mers in the 3′ UTRs of genes from highly expressed intestinal and neuronal genes. The analysis is also done for miRNAs that were enriched and statistically significant in neurons. D. Chart showing the average percent rank of highly expressed neuronal genes (from ref. 11) within a given dataset. A student's t-test was used to determine statistical significance.
(PDF)

Table S1 GO term enrichments. GO term enrichments from GOrilla for enriched processes, functions, and cellular components.
(XLSX)
