# Supplementary figures and images for: Functional Analysis of Neuronal MicroRNAs in Caenorhabditis elegans Dauer Formation by Combinational Genetics and Neuronal miRISC Immunoprecipitation
Source: PLoS Genet. 2013 Jun 20;9(6):e1003592. doi: 10.1371/journal.pgen.1003592 (PMC3688502; doi:10.1371/journal.pgen.1003592)

**A**

| Strain                      | % Dauer 25°C (n) |
|-----------------------------|------------------|
| <i>unc-3(lf)</i>            | 0 (462)          |
| <i>unc-3(lf); alg-2(lf)</i> | 0 (412)          |

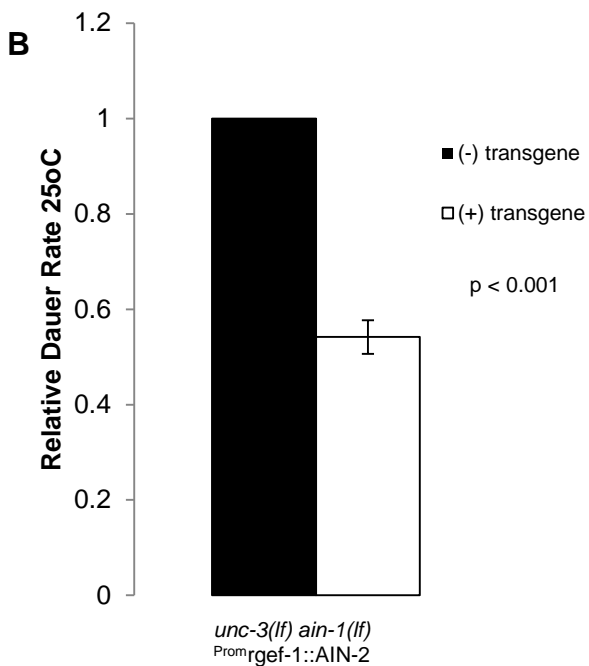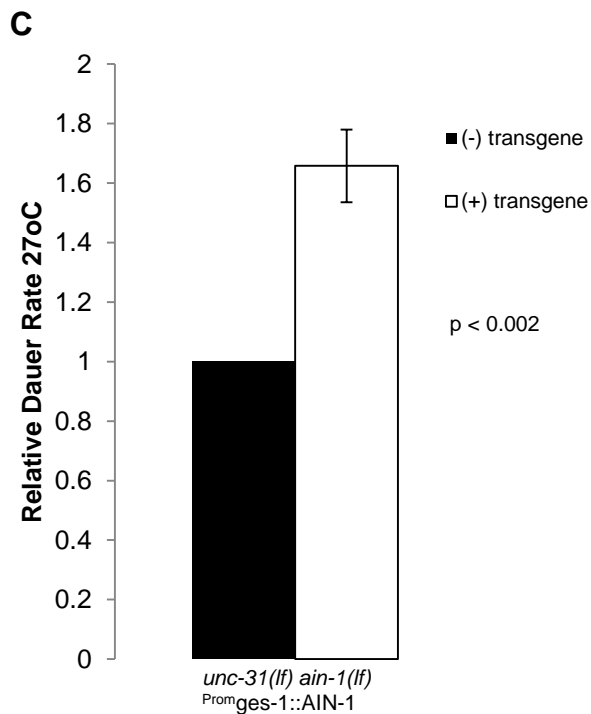

**D**

| Strain                      | % Dauer 27°C (n) |
|-----------------------------|------------------|
| <i>lim-4(lf)</i>            | 0 (493)          |
| <i>lim-4(lf); ain-1(lf)</i> | 0 (307)          |

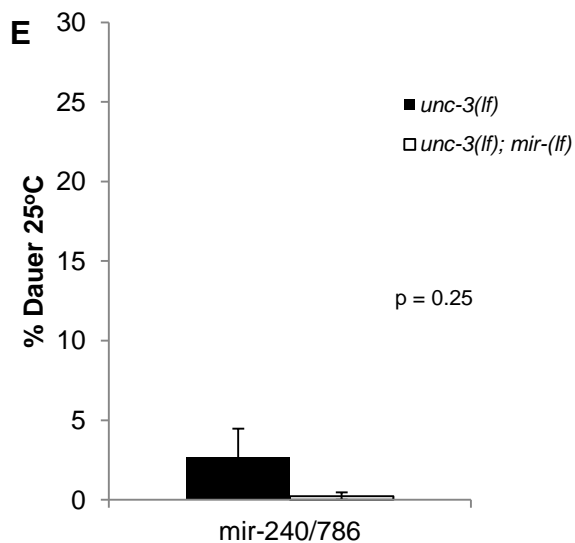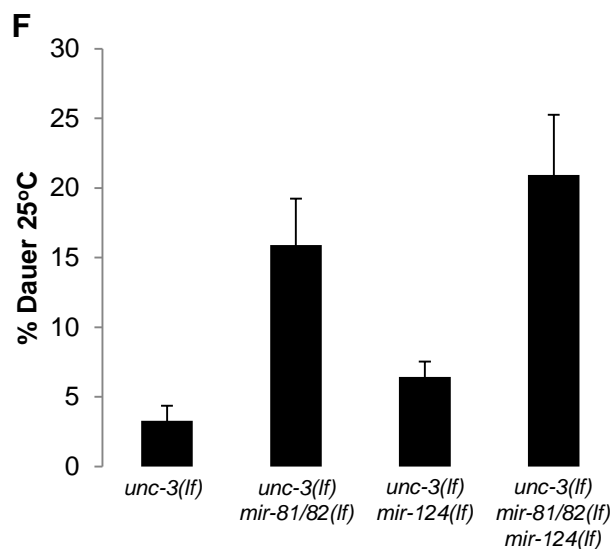

Supplement: Figure S1 — Additional dauer assays. A, D. Chart showing percentage of dauer of indicated strains, two biological replicates were done. B, C. Relative rate of dauer formation of indicated strains at indicated temperature. Data is the average of at least two independent lines. E, F. Rates of dauer formation of indicated strains. (PDF) [file pgen.1003592.s003.pdf]
