# Supplementary material for: Functional Analysis of Neuronal MicroRNAs in Caenorhabditis elegans Dauer Formation by Combinational Genetics and Neuronal miRISC Immunoprecipitation
Source: PLoS Genet. 2013 Jun 20;9(6):e1003592. doi: 10.1371/journal.pgen.1003592 (PMC3688502; doi:10.1371/journal.pgen.1003592)
Supplement: Figure S2 — qPCR of potential miRNA targets. Chart showing the relative log2 enrichment of indicated gene in ain-1(ku322 lf); ain-2(tm1863 rf) vs N2 from four biological replicates. *indicates only three biological replicates were done. (PDF) [file pgen.1003592.s004.pdf]

Gene Name	Relative Log2 enrichment of mRNA ain-1/2 vs N2 ± S.E.M	P-value of enrichment of qPCR	Enrichment in miRISC IPs (percent rank)
odr-2	0.80 ± 0.11	0.000316	0.999965
mec-9	1.11 ± 0.48	0.05925	0.985155
nlp-9	2.22 ± 0.24	0.0001	0.951384
syd-1	0.96 ± 0.22	0.005298	0.99767
npr-3	1.22 ± 0.36	0.01435	0.989958
dop-3	0.88 ± 0.11	0.000166	0.994395
ins-1*	1.84 ± 0.42	0.01229	0.941564
pde-3	1.02 ± 0.28	0.009972	0.903074
lev-1	0.60 ± 0.31	0.110551	0.99407
agt-2	-0.36 ± 0.14	0.04109	0.072582
