# Supplementary material for: Functional Analysis of Neuronal MicroRNAs in Caenorhabditis elegans Dauer Formation by Combinational Genetics and Neuronal miRISC Immunoprecipitation
Source: PLoS Genet. 2013 Jun 20;9(6):e1003592. doi: 10.1371/journal.pgen.1003592 (PMC3688502; doi:10.1371/journal.pgen.1003592)
Supplement: Figure S3 — Relevant to Figure 5. Neuronal genes are likely miRNA targets. A. Chart showing the overlap and enrichment of highly expressed genes in various tissues within the neuronal miRISC dataset. A student's t-test was used to determine differences in average percent rank against all testable genes (average rank of 0.48). P-values and expected values were calculated using a hypergeometric distribution. Percentages were calculated from 100*[(# genes in overlap)/(# genes associated with neuronal AIN-2)]. B. Chart showing median 3′ UTR length and percent 3′ UTRs with at least one perfect 7-mer binding site to all annotated miRNAs, methods for statistics are similar as before. C. Bar graph showing the relative seed density of perfect 7-mers in the 3′ UTRs of genes from highly expressed intestinal and neuronal genes. The analysis is also done for miRNAs that were enriched and statistically significant in neurons. D. Chart showing the average percent rank of highly expressed neuronal genes (from ref. 11) within a given dataset. A student's t-test was used to determine statistical significance. (PDF) [file pgen.1003592.s005.pdf]

A.

Dataset (ref)	Average percent rank (p val)	Genes in overlap (expected), p val	Percentage of neuronal AIN-2
Muscle (31)	0.51 (0.00014)	51 (49), 0.0556	7.0%
Intestine (32)	0.45 (1.7×10^{-5})	55 (70), 0.0080	7.6%
Neuron (12)	0.73 (5.5×10^{-241})	350 (64), 2.2×10^{-184}	48.1%

B.

Dataset (ref)	Median 3' UTR length (p val)	% 3' UTRs with at least 1 7-mer (p val)
All UTRs (33)	121	63.8%
Intestine (32)	121 (1)	64.4% (0.014)
Neuron (12)	165.5 (6.3×10^{-6})	75.1% (1.5×10^{-33})

C.

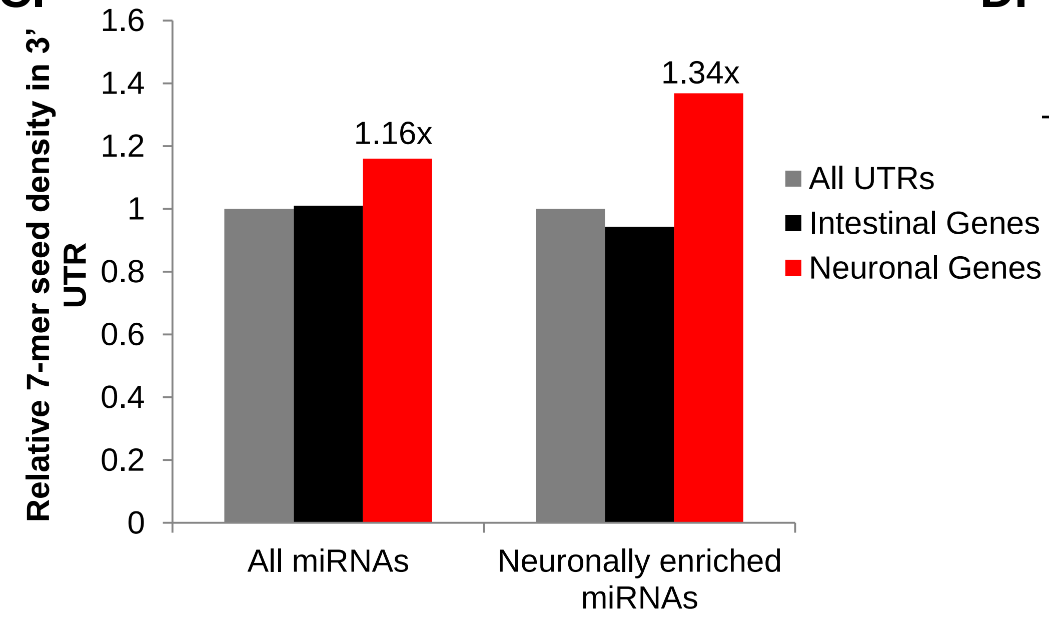

D.

Dataset (from ref 4)	Average Percent Rank of Dataset	Average percent rank of neuonal genes (pval)
GFP Control	0.50	0.52 (0.0022)
Asynchronous Intestine	0.50	0.53 (1.7×10^{-5})
Muscle	0.50	0.59 (1.9×10^{-34})
L4 Intestine	0.50	0.56 (1.1×10^{-13})
